# Role of the Multidrug Resistance Efflux Pump MexCD-OprJ in the *Pseudomonas aeruginosa* Quorum Sensing Response

**DOI:** 10.3389/fmicb.2018.02752

**Published:** 2018-11-23

**Authors:** Manuel Alcalde-Rico, Jorge Olivares-Pacheco, Carolina Alvarez-Ortega, Miguel Cámara, José Luis Martínez

**Affiliations:** ^1^Centro Nacional de Biotecnología, Consejo Superior de Investigaciones Científicas, Madrid, Spain; ^2^Centre for Biomolecular Sciences, School of Life Sciences, University of Nottingham, Nottingham, United Kingdom

**Keywords:** *Pseudomonas aeruginosa*, quorum sensing, antibiotic resistance, multidrug efflux pump, MexCD-OprJ

## Abstract

Multidrug efflux pumps constitute a category of antibiotic resistance determinants that are a part of the core bacterial genomes. Given their conservation, it is conceivable that they present functions beyond the extrusion of antibiotics currently used for therapy. *Pseudomonas aeruginosa* stands as a relevant respiratory pathogen, with a high prevalence at hospitals and in cystic fibrosis patients. Part of its success relies on its low susceptibility to antibiotics and on the production of virulence factors, whose expression is regulated in several cases by quorum sensing (QS). We found that overexpression of the MexCD-OprJ multidrug efflux pump shuts down the *P. aeruginosa* QS response. Our results support that MexCD-OprJ extrudes kynurenine, a precursor of the alkyl-quinolone signal (AQS) molecules. Anthranilate and octanoate, also AQS precursors, do not seem to be extruded by MexCD-OprJ. Kynurenine extrusion is not sufficient to reduce the QS response in a mutant overexpressing this efflux pump. Impaired QS response is mainly due to the extrusion of 4-hydroxy-2-heptylquinoline (HHQ), the precursor of the *Pseudomonas* Quinolone Signal (PQS), leading to low PQS intracellular levels and reduced production of QS signal molecules. As the consequence, the expression of QS-regulated genes is impaired and the production of QS-regulated virulence factors strongly decreases in a MexCD-OprN *P. aeruginosa* overexpressing mutant. Previous work showed that MexEF-OprJ, another *P. aeruginosa* efflux pump, is also able of extruding kynurenine and HHQ. However, opposite to our findings, the QS defect in a MexEF-OprN overproducer is due to kynurenine extrusion. These results indicate that, although efflux pumps can share some substrates, the affinity for each of them can be different. Although the QS response is triggered by population density, information on additional elements able of modulating such response is still scarce. This is particularly important in the case of *P. aeruginosa* lung chronic infections, a situation in which QS-defective mutants are accumulated. If MexCD-OprJ overexpression alleviates the cost associated to triggering the QS response when un-needed, it could be possible that MexCD-OprJ antibiotic resistant overproducer strains might be selected even in the absence of antibiotic selective pressure, acting as antibiotic resistant cheaters in heterogeneous *P. aeruginosa* populations.

## Introduction

*Pseudomonas aeruginosa* is a free-living microorganism able to survive in different environments that not only plays an ecological role in natural ecosystems (Green et al., 1974; Romling et al., 1994; Morales et al., 2004; Walker et al., 2004), but it is also an important causative agent of infections in patients with underlying diseases (Obritsch et al., 2005; Driscoll et al., 2007; Martinez-Solano et al., 2008; Talwalkar and Murray, 2016). The characteristic low susceptibility to antibiotics of this organism relies on several factors (Fajardo et al., 2008). Particularly relevant is the activity of chromosomally-encoded multidrug resistance (MDR) efflux pumps (Vila and Martínez, 2008; Hernando-Amado et al., 2016). Further, the acquisition of mutation-driven resistance is common in this opportunistic pathogen, particularly along chronic infections (Oliver et al., 2015; Palmer and Whiteley, 2015; Lopez-Causape et al., 2018), where the constitutive overexpression of MDR efflux pumps is one of the biggest problems to eradicate these infections (Vila and Martínez, 2008; Vila et al., 2011; Hernando-Amado et al., 2016). Efflux pumps exhibit different functions, with physiological and ecological significances that go beyond their activity as antibiotic resistance elements (Piddock, 2006; Martinez et al., 2009; Alcalde-Rico et al., 2016; Hernando-Amado et al., 2016). In the case of *P. aeruginosa*, an opportunistic pathogen not fully adapted to human hosts (Alonso et al., 1999; Morales et al., 2004), these functions should be of relevance for the success of *P. aeruginosa* as a respiratory infectious pathogen.

*Pseudomonas aeruginosa* harbors several efflux systems that belong to different families (Stover et al., 2000). The most studied because of their clinical relevance are MexAB-OprM (Li et al., 1995), MexCD-OprJ (Poole et al., 1996; Kohler et al., 1997), MexEF-OprN (Kohler et al., 1997), and MexXY (Aires et al., 1999; Mine et al., 1999). They all belong to the *Resistance-Nodulation-Division* (RND) family of MDR systems (Hernando-Amado et al., 2016). Mutants that exhibit constitutive overexpression of each of these efflux pumps are selected upon treatment with antibiotics; the mutations are frequently located in the regulatory elements adjacent to the respective operon encoding for these MDR systems (Llanes et al., 2004; Sobel et al., 2005; Jeannot et al., 2008; Zaborskyte et al., 2017).

The unregulated overexpression of an efflux system not only contributes to antibiotic resistance but may also have pleiotropic effects in the bacterial physiology. We have recently reported that overexpression of RND systems in *P. aeruginosa* leads to an excessive internalization of protons that acidify the cytoplasm, which causes a biological cost in absence of oxygen or nitrate, since both are necessary to compensate for the intracellular H^+^ accumulation (Olivares et al., 2014; Olivares Pacheco et al., 2017). In addition to these non-specific effects, other effects might be due to the unregulated extrusion of intracellular compounds, some of which may be relevant for the ecological behavior of *P. aeruginosa* (Blanco et al., 2016). Indeed, different studies have shown that overexpression of MDR efflux pumps may challenge the *P. aeruginosa* quorum sensing (QS) response (Evans et al., 1998; Kohler et al., 2001; Linares et al., 2005; Olivares et al., 2012), which is in turn determinant for modulating several physiological processes, including bacterial pathogenicity, in response to population density (Williams et al., 2007).

In *P. aeruginosa*, the QS-signaling network consists of three main interconnected regulatory systems: Las, Rhl, and Pqs, which synthetize and respond to the autoinducers *N*-(3-oxododecanoyl)-L-homoserine lactone (3-oxo-C12-HSL), *N*-butanoyl-L-homoserine lactone (C4-HSL), and the 2-alkyl-4(1*H*)-quinolones (AQs) *Pseudomonas* Quinolone Signal (PQS, or its immediate precursor 2-heptyl-4-hydroxyquinoline, HHQ), respectively (Williams and Camara, 2009). These autoinducers are able to bind to their respective transcriptional regulators, namely LasR, RhlR and PqsR, thus controlling the expression of a large number of genes including those responsible for their own synthesis: *lasI*, *rhlI*, and *pqsABCDE*, respectively.

Some *P. aeruginosa* RND systems have been associated with QS. MexAB-OprM is induced by C4-HSL (Maseda et al., 2004) and has been proposed to extrude 3-oxo-C12-HSL and other 3-oxo-HSL related compounds (Evans et al., 1998; Pearson et al., 1999; Minagawa et al., 2012). MexEF-OprN is able to efflux HHQ (Lamarche and Déziel, 2011) and kynurenine (Olivares et al., 2012), both precursors of the PQS autoinducer signal (Farrow and Pesci, 2007; Palmer et al., 2013). In agreement with these findings, the antibiotic resistant mutants that overproduce MexAB-OprM or MexEF-OprN have been associated with a low production of QS-controlled virulence factors (Evans et al., 1998; Pearson et al., 1999; Sanchez et al., 2002; Olivares et al., 2012). Some studies have demonstrated that acquisition of antibiotic resistance due to constitutive overexpression of *mexCD-oprJ* correlates with a decrease in the production of several virulence factors, some of them controlled by QS (Sanchez et al., 2002; Linares et al., 2005; Jeannot et al., 2008; Stickland et al., 2010). However, the underlying reasons for this correlation remain to be elucidated. In this work, we analyzed in depth the production of each QS signal molecule (QSSM) and the expression levels of the genes controlled by these regulation systems in order to understand how overexpression of MexCD-OprJ could be affecting the *P. aeruginosa* QS response, and consequently the behavior of this bacterial pathogen in the infected patient.

## Materials and Methods

### Bacterial Strains, Plasmids, Primers, and Culture Conditions

The *Escherichia coli* and *P. aeruginosa* strains and the plasmids used in this work, are listed in the Table 1. The primers used are listed in the Table 2.

**Table 1:** Bacterial strains and plasmids used in the present work.

Bacterial strain/plasmids	Description	Reference/origin
*Escherichia coli*		
One Shot OmniMax^TM^ 2 T1	Host strain used for the maintenance of cloning plasmids: F′ *pro*AB *lac*I^q^ *lac*ZΔM15 *Tn*10(Tet^R^) Δ(*ccd*AB) *mcr*A, Δ(*mrr*,*hsd*RMS-*mcr*BC) ϕ80(*lac*Z)ΔM15 Δ(*lac*ZYA-*arg*F)U169 *end*A1 recA1 *sup*E44 *thi*-1 *gyr*A96 *rel*A1 *ton*A *pan*D	Invitrogen
S17-1λ *pir*	Conjugative donor strain used for transferring plasmids to *P. aeruginosa* acceptor strains by conjugation assays: F^−^ *thi* *pro* *hsdR* *hsdM*^+^ *recA* RP42-Tc::Mu-Km::Tn7	Simon et al., 1986
S17 miniCTX::P*pqsA-lux*	S17-1λ *pir* strain containing the miniCTX::P*pqsA-lux* plasmid	Fletcher et al., 2007a,b
JM109-pSB1142 (LasR-based Biosensor)	Biosensor strain used for detecting the QS signal, 3-oxo-C12-HSL, produced by *P. aeruginosa* strains	Winson et al., 1998
JM109-pSB536 (RhlR-based Biosensor)	Biosensor strain used for detecting the QS signal, C4-HSL, produced by *P. aeruginosa* strains	Swift et al., 1997
*Pseudomonas aeruginosa*		
PAO1	Wild-type PAO1-V clinic strain given from the lab of V. de Lorenzo	Linares et al., 2005
PAO1 miniCTX::P*pqsA-lux* (PAO1_P*pqsA*)	PAO1-V strain with the reporter construction P*pqsA-luxCDABE* inserted in the specific *attB* site of the chromosome	Present work
JFL28 (*nfxB*^∗^)	Spontaneous resistant mutant obtained from PAO1-V strain, which overproduces the MexCD-OprJ efflux system by punctual inactivating mutation in *nfxB* gene	Linares et al., 2005
JFL28 miniCTX::P*pqsA-lux* (*nfxB^∗^*_P*pqsA*)	JFL28 strain with the reporter construction P*pqsA-luxCDABE* inserted in the specific *attB* site of the chromosome	Present work
*nfxB*^∗^Δ*mexD*	JFL28 strain with an inactive MexCD-OprJ efflux system by partial deletion of the *mexD* gene	Present work
PAO1 CTX::P*_pqsA_-lux::pqsA* (PqsR-based Biosensor)	PAO1-Δ*pqsA* strain with the reporter construction P*pqsA-luxCDABE* inserted in the specific *attB* site of the chromosome. Used for detecting the AQs produced by other *P. aeruginosa* strains	Fletcher et al., 2007a,b
Plasmid		
pGEM-T Easy	Commercial plasmid used for cloning optimization of PCR products (Amp^R^)	Promega
pGEM-T-Δ*mexD*	pGEM-T Easy vector with the flanking DNA sequences of a 2058 bp inner region of *mexD* gene (Amp^R^)	Present work
pEX18Ap	Plasmid with conjugative properties used for deleting genes in *P. aeruginosa* by homolog recombination. Amp^R^	Hoang et al., 1998
pEX18Ap-Δ*mexD*	pEX18Ap vector with the flanking DNA sequences of a 2058 bp inner region of *mexD* gene used for deleting *mexD* gene in *P. aeruginosa* strains. Amp^R^	Present work
Mini-CTX-*lux*-P*pqsA*	Plasmid derived from mini-CTX-*lux* Becher and Schweizer (2000) in which the expression of the *luxCDABE* operon is under the transcriptional control of the *pqsABCDE* promoter region of *P. aeruginosa*. Tc^R^	Fletcher et al., 2007a,b
pSB1142	Plasmid carried by the LasR-Bioreporter strain necessary for detecting 3-oxo-C12-HSL. Tc^R^	Winson et al., 1998
pSB536	Plasmid carried by the RhlR-Bioreporter strain necessary for detecting C4-HSL. Amp^R^	Swift et al., 1997

**Table 2:** Collection of primers used in the present work.

Name	Sequence	Description
HindIII_*mexD*_Fw *mexD*_int_Rev	5′-CCCAAGCTTCGAGGTGCGCGCGCGGGTGGCCGGC-3′	Amplification of the DNA flanking region “Up” for deleting *mexD* gene
	5′-GCGAGCCTGCAGCAGCGCTTATTCGGACATCGGλATCC-3′	
*mexD*_int_Fw HindIII_*mexD*_Rev	5′-GGATTTTCCGATGTCCGAATAAGCGCTGCTGCAGGCTCGC-3′	Amplification of the DNA flanking region “Down” for deleting *mexD* gene
	5″-CCCAAGCTTCAGACGλCAGATAGGTACGAACA-3′	
Δ*mexD*_check_Fw Δ*mexD*_check_Rev	5′-GGTGAAGATCGTGCCGAAG-3′	To check the deletion of the *mexD* gene
	5′-ATTGGTGAAGTCGTTGATCA-3′	
M13_Fw M13_Rev	5′-CACGACGTTGTλACGAC-3′	To check the insertion of cloning DNA fragment into pGEM-t Easy vector
	5′-GGATAACAATTTCACACAGG-3′	
*rplU*_Fwd *rplU*_Rev	5′-CGCAGTGATTGTTACCGGTG-3′	To check DNA contamination of RNA samples
	5′-AGGCCTGAATGCCGGTGATC-3′	
*rpsL*_Fwd *rpsL_*Rev	5′-GCAAGCGCATGGTCGACAAGA-3′	Real-time RT-PCR (Housekeeping)
	5′-CGCTGTGCTCTTGCAGGTTGTGA-3′	
*lasA*_Fwd *lasA*_Rev	5′-ATGGACCAGATCCAGGTGAG-3′	Real-time RT-PCR
	5′-CGTTGTCGTAGTTGCTGGTG-3′	
*lasB_*Fwd *lasB*_Rev	5′-ATCGGCAAGTACACCTACGG-3′	Real-time RT-PCR
	5′-ACCAGTCCCGGTACAGTTTG-3′	
*rhlA*_Fwd *rhlA_*Rev	5′-CGAGGTCAATCACCTGGTCT-3′	Real-time RT-PCR
	5′-GACGGTCTCGTTGAGCAGAT-3′	
*rhlB*_Fwd *rhlB*_Rev	5′-GAGCGACGAACTGACCTACC-3′	Real-time RT-PCR
	5′-GGGAATCCCGTACTTCTCGT-3′	
*lecA_*Fwd *lecA*_Rev	5′-ATAACGAAGCAGGGCAGGTA-3′	Real-time RT-PCR
	5′-TTGCCAATCTTCATGACCAG-3′	
*phzB1*_Fwd *phzB1_*Rev	5′-AACGAACTTCGCGλAGAA-3′	Real-time RT-PCR
	5′-TTTGTCTTTGCCACGAATGA-3′	
*phzB2*_Fwd *phzB2*_Rev	5′-GCGAGACGGTGGTCAAGTAT-3′	Real-time RT-PCR
	5′-AATCCGGGAAGCATTTCAG-3′	
*phzS_*Fwd *phzS*_Rev	5′-CAAGTCGCTGGTGAACTGG-3′	Real-time RT-PCR
	5′-CGGGTACTGCAGGATCAACT-3′	
*mexG*_Fwd *mexG_*Rev	5′-GGCGAAGCTGTTCGACTATC-3′	Real-time RT-PCR
	5′-AGAAGGTGTGGACGATGAGG-3′	
*lasI*_Fwd *lasI*_Rev	5′-CTACAGCCTGCAGAACGACA-3′	Real-time RT-PCR
	5′-ATCTGGGTCTTGGCATTGAG-3′	
*rhlI_*Fwd *rhlI*_Rev	5′-CTCTCTGAATCGCTGGAAGG-3′	Real-time RT-PCR
	5′-GACGTCCTTGAGCAGGTAGG-3′	
*pqsA*_Fwd *pqsA_*Rev	5′-CAATACACCTCGGGTTCCAC-3′	Real-time RT-PCR
	5′-TGAACCAGGGλGAACAGG-3′	
*pqsD*_Fwd *pqsD*_Rev	5′-CATGTGATCTGCCATCAACC-3′	Real-time RT-PCR
	5′-AGCCGTAGGTCAGGACCAG-3′	
*pqsE*_Fwd *pqsE*_Rev	5′-GACATGGAGGCTTACCTGGA-3′	Real-time RT-PCR
	5′-CTCAGTTCGTCGAGGGATTC-3′	
*phnB_*Fwd *phnB*_Rev	5′-CACTCGCTGGTGGTCAGTC-3′	Real-time RT-PCR
	5′-AGAGTAGAGCGTTCTCCAGCA-3′	
*pqsH*_Fwd *pqsH_*Rev	5′-ATGTCTACGCGACCCTGAAG-3′	Real-time RT-PCR
	5′-AACTCCTCGAGGTCGTTGTG-3′	

Unless other conditions are specified, experiments were carried out at 37°C in 100 ml flasks containing 25 ml of LB broth (Lennox). The *E. coli* strains carrying plasmids with ampicillin (Amp^R^) or tetracycline (Tc^R^) resistance genes were grown in LB medium with 100 μg/ml of ampicillin or 10 μg/ml of tetracycline, respectively. For determining the effect of different carbon sources on *P. aeruginosa* growth, overnight cultures were washed with M63 medium containing MgSO_4_ 1 mM and diluted to an OD_600_ = 0.01 in clear bottom 96-well plates containing 150 μl/well of M63 with the corresponding carbon source at a final concentration of 10 mM. The growth of each strain was measured at 37°C using a multi-plate reader.

### Whole Genome Sequence of the *nfxB^∗^* Strain and Generation of a *nfxB^∗^*Δ*mexD* Mutant

The *nfxB^∗^* mutant was fully sequenced at Parque Científico de Madrid using Illumina technology as described (Garcia-Leon et al., 2014). Two ≈1000 bp DNA regions adjacent to the fragment of *mexD* to be deleted were amplified by PCR using the primers listed in Table 2. The amplicons were purified and used together for a nested PCR reaction in which a recombinant 2058 bp DNA was generated and cloned into pGEM-t Easy (pGEM-T-Δ*mexD*). *E. coli* OmniMax^TM^ cells were transformed with this plasmid and the sequence of the construction was verified by Sanger sequencing. The fragment was excised using HindIII and subcloned into pEX18Ap. The resulting pEX18Ap-Δ*mexD* construction was incorporated into *E. coli* S17-1λ *pir* by transformation. Introduction of the deleted allele into *P. aeruginosa* *nfxB*^∗^ was performed by conjugation using S17-1λ *pir* (pEX18Ap-Δ*mexD*) as donor strain as described (Hoang et al., 1998). *mexD* deletion was confirmed by PCR using the primers described in Table 2.

### Analysis of the Production of QS-Regulated Virulence Factors

The secretion of elastase and protease IV was measured after 20 h of incubation of the bacterial cultures in LB at 37°C following the methods described in Kessler and Safrin (2014). Rhamnolipids detection was carried out as described (Wittgens et al., 2011). Pyocyanin was determined as detailed (Essar et al., 1990). For the swarming motility assay, O/N cultures were washed with sterile 0.85% NaCl and diluted to an OD_600_ = 1.0. Five-microliters drops were poured on the center of Petri dishes containing 25 ml of a defined medium (0.5% casamino acids, 0.5% bacto agar, 0.5% glucose, 3.3 mM K_2_HPO_4_, and 3 mM MgSO_4_), which were incubated 16 h at 37°C.

### RNA Extraction and Real-Time RT-PCR

RNA was obtained using the RNeasy mini kit (QIAGEN) as described (Olivares et al., 2012). After treatment with DNase (Olivares et al., 2012), the presence of DNA contamination was checked by PCR using *rplU* primers. Real-time RT-PCR was performed as described in Olivares et al. (2012) using the primers listed in Table 2. The experiments were carried out in triplicate. The 2^−ΔΔCt^ method (Livak and Schmittgen, 2001) was used for quantifying the results, normalizing the results to the housekeeping gene, *rpsL*.

### Thin Layer Chromatography (TLC) and Time Course Monitoring of QSSMs Accumulation

Bacterial O/N cultures were washed with fresh LB medium and diluted to an OD_600_ = 0.01 for subsequent growth. For TLC assays, the QSSMs extractions were carried out as described (Fletcher et al., 2007a). For time course assays, this protocol was optimized to simultaneous monitoring QSSMs accumulation and cell density. For each extraction time, 1.8 ml aliquots from cultures were centrifuged (7,000 × *g*, 10 min at 4°C). The supernatants were filtered through 0.22 μm pore size membrane and the cellular pellets were resuspended in 1.8 ml of methanol HPLC grade to extract the QSSMs. 900 μl of cell-free supernatants were used to extract the QSSMs by adding 600 μl of acidified ethyl acetate twice. The resulting acidified ethyl acetate extracts were dried and subsequently dissolved in 900 μl of methanol HPLC grade.

Alkyl-quinolone signal (AQs) were detected by TLC as described (Fletcher et al., 2007a) using the PAO1 CTX::P*_pqsA_-lux* biosensor strain. C4-HSL and 3-oxo-C12-HSL were analyzed using the JM109-pSB536 (RhlR- based biosensor) and JM109-pSB1142 (LasR-based biosensor) biosensor strains, respectively (Yates et al., 2002). The image processing software “ImageJ” was used for densitometry analysis of the light spots.

For time course accumulation assays, flat white 96-well plates with optical bottom were filled with a mix containing 5 μl of sample and 195 μl of a 1/100 dilution of the corresponding O/N biosensor cultures. The experiments were carried out on a multi-plate luminometer/spectrophotometer reader. The highest relative light units (RLU = luminescence/OD_600_ ratio) obtained for each biosensor strain and the OD_600_ in which the samples were taken from *P. aeruginosa* cultures were represented.

### Analysis by HPLC-MS of Kynurenine and Anthranilate Accumulation in Cell-Free Supernatants

Bacterial strains were grown in M63 containing succinate (10 mM) and tryptophan (10 mM). After 24 h at 37°C, the supernatants were filtered through a 0.22 μm pore size membrane and lyophilized. 100 mg of each sample were resuspended in 2 ml of 3 mM ammonium acetate and dissolved in H_2_O/methanol (50/50). The amounts of anthranilate and kynurenine were determined by HPLC-MS at Laboratorio de Cromatografía-SIdI from the Universidad Autónoma de Madrid.

### Insertion of the Reporter Construction, miniCTX::P*pqsA-lux*, in the Chromosome of *P. aeruginosa* and Analysis of *pqsABCDE* Expression

The insertion of the miniCTX::P*pqsA-lux* reporter into the chromosomes of the different *P. aeruginosa* strains was carried out by conjugation as described (Hoang et al., 1998) using *E. coli* S17-1λ *pir* containing miniCTX::P*pqsA*-*lux* (Fletcher et al., 2007b) as donor strain. The resulting *P. aeruginosa* reporter strains were inoculated in flat white 96-well plates with optical bottom containing 200 μL of LB with or without 4 mM anthranilate at an initial OD_600_ = 0.01. The growth (OD_600_) and the bioluminescence emitted by the P*pqsA-luxCDABE* construction was monitored using a multi-plate reader.

### Statistical Analysis

At least three biological replicates were analyzed in each experiment. Statistical significance was evaluated by using a Student’s two-tailed test with a confidence interval of 95%. The differences were considered significant for *P*-values < 0.05 (^∗^*P* < 0.05; ^∗∗^*P* < 0.01; ^∗∗∗^*P* < 0.001). The quantification of the areas under the curves was carried out using the GraphPad Prim software and the mean of each biological replicates were used to calculate statistical significance.

## Results

Increased expression of efflux pumps due to mutations in their regulators can produce different changes in bacterial physiology. In most cases, the phenotypes observed in this kind of mutants are attributed to the activity of the overexpressed efflux pump. However, in other instances, the mutations in the local regulator itself might have effects on the bacterial physiology, which affect bacterial virulence and are independent of the activity of the efflux pump (Tian et al., 2009a,b). To address this possibility, we used a previously described mutant that overexpresses MexCD-OprJ (Linares et al., 2005). To discard the possibility that other mutations besides those in the *mexCD-oprJ* repressors might have been selected in this strain during its stay in the laboratory, the genome of the mutant was fully sequenced. Only the already described *nfxB* mutation (Linares et al., 2005) was found. From this mutant, an *nfxB*^∗^Δ*mexD* strain, which keeps the *nfxB* mutation in addition to a partial deletion of the *mexD* gene, was generated. By comparing *nfxB^∗^* and *nfxB*^∗^Δ*mexD* strains, we were able to define more precisely which phenotypes depend on the activity of the efflux pump and which are solely due to the inactivation of the NfxB repressor, independently of the activity of the efflux pump.

### Overexpression of MexCD-OprJ Results in a Decrease in the Production of QS-Controlled Virulence Factors in *P. aeruginosa*

**FIGURE 1:**
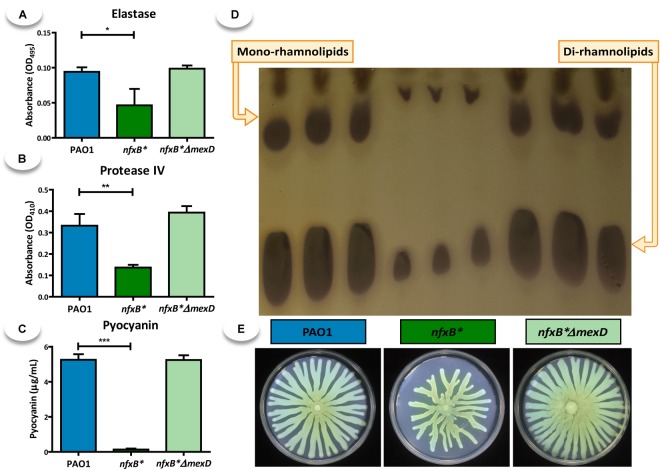
Overexpression of the MexCD-OprJ efflux pump results in a decrease in the production of different virulence factors regulated by the QS system. The elastase **(A)**, protease IV **(B)**, pyocyanin **(C)**, rhamnolipids, **(D)** and swarming **(E)** assays were conducted as described in Methods using cultures of the PAO1, *nfxB^∗^* and *nfxB*^∗^Δ*mexD* strains. Statistical significances were evaluated by using a Student’s two-tailed test and considered significant if *P* < 0.05, with a confidence interval of 95% (^∗^*P* < 0.05; ^∗∗^*P* < 0.01; ^∗∗∗^*P* < 0.001). *nfxB^∗^* presented a lower production of all tested virulence factors that the parental wild-type PAO1. The deletion of *mexD* in strain *nfxB^∗^mexD* restores the phenotypes to the levels of the wild-type strain, indicating that the defects in the expression of virulence factors were solely due to the activity of the *mexCD-oprJ* efflux pump.

Swarming motility and the production of elastase, proteinase IV, pyocyanin, and, rhamnolipids were analyzed to establish whether or not MexCD-OprJ affects the production of *P. aeruginosa* QS-regulated virulence elements. As Figure 1 shows and in agreement with previous studies (Sanchez et al., 2002; Stickland et al., 2010), the *nfxB^∗^* strain exhibits a decrease in swarming motility and in the production of all analyzed virulence factors in comparison with the wild-type PAO1 strain. The fact that the deletion of *mexD* fully restores the production of QS-regulated virulence factors in an *nfxB^∗^* background, indicates that the observed impairment is solely due to the activity of MexCD-OprJ, independently of the potential activity of the NfxB regulator protein.

### Overproduction of the MexCD-OprJ Efflux System Results in a Lower Expression of QS- Regulated Genes

**FIGURE 2:**
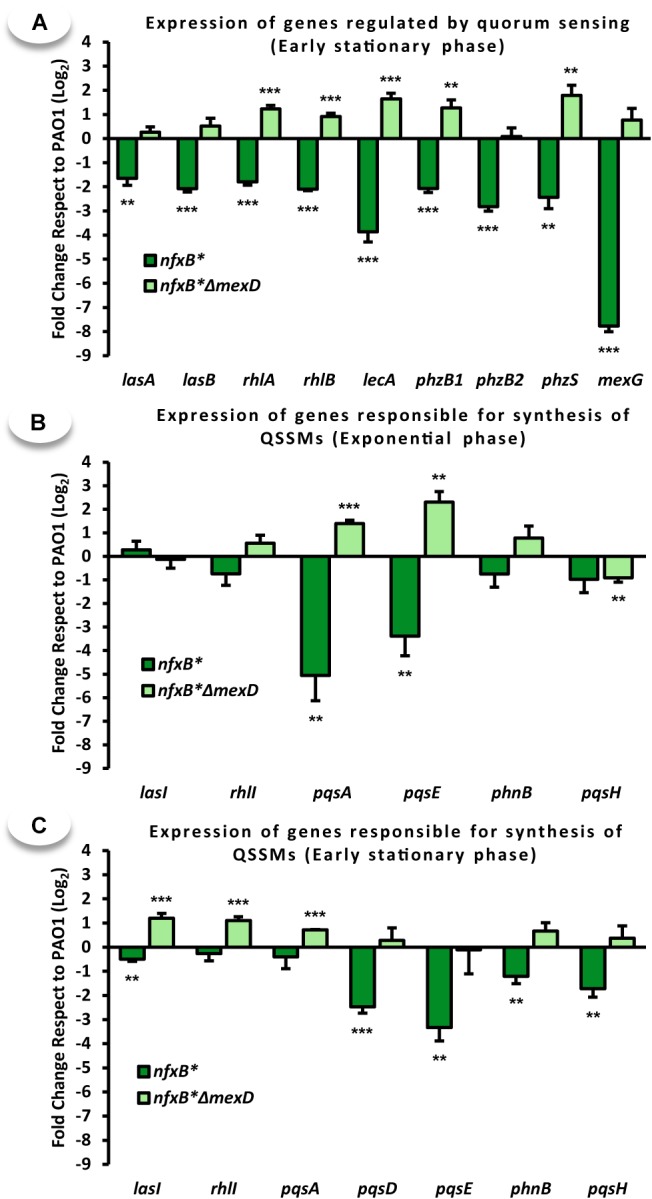
Overexpression of the MexCD-OprJ efflux system affects the expression levels of QS-regulated genes. Transcriptional analysis by real-time RT-PCR of **(A)** genes regulated by quorum sensing (QS) response (*lasA*, *lasB*, *rhlA*, *rhlB*, *lecA*, *phzB1*, *phzB2*, *phzS*, and *mexG*) and **(B,C)** genes responsible for QSSMs production (*lasI*, *rhlI*, *pqsA*, *pqsD*, *pqsE*, *phnB*, and *pqsH*) from samples obtained in **(B)** exponential (OD_600_ = 0.6) and **(A,C)** early stationary phase of growth (OD_600_ = 2.5) in PAO1, *nfxB^∗^* and *nfxB*^∗^Δ*mexD* strains grown in LB medium. Statistical significances were evaluated by using a Student’s two-tailed test and considered significant if *P* < 0.05, with a confidence interval of 95% (^∗^*P* < 0.05; ^∗∗^*P* < 0.01; ^∗∗∗^*P* < 0.001). The results showed that the genes implicated in the synthesis of PQS *pqsA* and *pqsE* were expressed at lower level in the *nfxB*^∗^ strain than in the wild-type PAO1 strain at exponential phase of growth **(B)**. In early stationary growth phase **(A,C)**, the expression levels of the PQS-biosynthesis genes (*pqsD*, *pqsE*, *phnB*, and *pqsH*), as well as all of the analyzed QS-regulated genes, were significantly lower in the *nfxB*^∗^ strain than in the PAO1 wild-type. The deletion of *mexD* in strain *nfxB^∗^mexD* restores or even increase the levels of expression to those of the wild-type strain, indicating that these defects were solely due to the activity of the *MexCD-OprJ* efflux pump.

Expression of a set of QS-regulated genes (Pearson et al., 1997; Deziel et al., 2003, 2005; Dietrich et al., 2006; Rampioni et al., 2016) was analyzed to determine if a low production of virulence factors in the *nfxB^∗^* mutant correlates with a deregulated expression of QS-regulated genes. LasB controls elastase production (Pearson et al., 1997; Kessler and Safrin, 2014). RhlA and RhlB are implicated in rhamnolipids biosynthesis (Pearson et al., 1997; Deziel et al., 2003), which in turn is important for swarming motility (Deziel et al., 2003). PhzB1, PhzB2, and PhzS are implicated in pyocyanin biosynthesis and the MexGHI-OpmD efflux pump has been described to be regulated by this phenazine (Dietrich et al., 2006). As shown in Figure 2A, the expression levels of the tested genes are lower in the *nfxB^∗^* strain than in PAO1. In addition, the expression of these genes is restored to PAO1 levels, even overcoming them, upon *mexD* deletion in the *nfxB^∗^* strain, further confirming that *mexCD-oprJ* overexpression is what causes an impaired QS response in the *nfxB^∗^* mutant. These results are in agreement with the lower production of virulence factors observed in *nfxB^∗^* (Figure 1).

To gain more insights on the reasons for this impaired QS-response, we analyzed the expression of genes responsible for the production of both families of autoinducers AHLs (*lasI* and *rhlI*) (Pesci et al., 1997) and AQs (*pqsABCDE*-*phnAB* and *pqsH*) (Gallagher et al., 2002). This was performed along the exponential growth phase when expression of these QS biosynthesis genes starts, and in early stationary phase, when the Pqs-system is fully active (Lepine et al., 2003). As shown in Figures 2B,C, expression of the genes responsible for the synthesis of PQS and HHQ exhibit a marked decrease in the *nfxB*^∗^ strain at both time points. These changes were restored to wild-type levels upon MexCD-OprJ inactivation in an *nfxB^∗^* background. *pqsA*, from the *pqsABCDE* operon responsible for the biosynthesis of AQs (Gallagher et al., 2002), exhibits the sharpest decrease in expression during exponential growth phase (Figure 2B). Expression of *phnB*, implicated in the synthesis of anthranilate through the chorismic acid pathway (Farrow and Pesci, 2007; Palmer et al., 2013), as well as *pqsH*, which codify the enzyme responsible for the conversion of HHQ into PQS (Gallagher et al., 2002), decreases more in early stationary phase (Figures 2B,C).

In contrast to the strong variations in expression of PQS-related genes, the activity of MexCD-OprJ had a minor impact on the expression of AHLs-related genes in both exponential and stationary growth phases. The *nfxB*^∗^ strain did not present alterations in *rhlI* expression, the gene responsible for the synthesis of C4-HSL, neither in exponential (Figure 2B) nor in stationary phase of growth (Figure 2C). A similar behavior was observed for *lasI*, the gene responsible for the synthesis of 3-oxo-C12-HSL, detecting just a slight decreased expression in the *nfxB*^∗^ strain during early stationary growth phase (Figures 2B,C).

### MexCD-OprJ Overexpression Entails a Decrease in the Production and Accumulation of AQs Due to Their Extrusion Through This Efflux Pump

**FIGURE 3:**
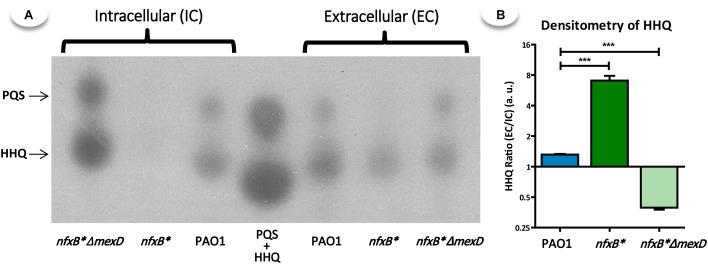
PQS and HHQ production is impaired in the strain that overproduces the MexCD-OprJ efflux pump. **(A)** To determine the accumulation levels of the autoinducers synthesized by *Pseudomonas aeruginosa*, a technique based on TLC coupled with a PqsR-based biosensor was used. The samples were extracted from cultures in early stationary phase (OD_600_ = 2.5). **(B)** The TLC-spots corresponding to HHQ were quantified by densitometry and the ratio between the HHQ present in the supernatant respect to cell extract was calculated and represented. Statistical significances were evaluated by using a Student’s two-tailed test and considered significant if *P* < 0.05, with a confidence interval of 95% (^∗^*P* < 0.05; ^∗∗^*P* < 0.01; ^∗∗∗^*P* < 0.001). As shown, overexpression of the MexCD-OprJ efflux system in *nfxB*^∗^ strongly reduces the production of PQS and HHQ as compared with PAO1 and *nfxB*^∗^Δ*mexD* strains. Furthermore, the analysis by densitometry of the HHQ ratio shows that this defect in AQs production is likely caused by an excessive extrusion of HHQ through MexCD-OprJ.

The production and accumulation of PQS and HHQ in both supernatant and cellular extracts decreased in the *nfxB^∗^* mutant (Figure 3A). This effect is directly dependent on MexCD-OprJ activity, since PQS/HHQ accumulation increased in the *nfxB*^∗^Δ*mexD* strain, even overcoming the wild-type levels in cell extracts (Figure 3A). Interestingly, the proportion of HHQ present in the supernatants with respect to cell-extracts is different among the three strains. As Figure 3B shows, the *nfxB^∗^* mutant has a higher supernatant/cell extract HHQ ratio than PAO1. Further, the deletion of *mexD* in the *nfxB^∗^* strain produced the opposite effect, decreasing the HHQ ratio to lower values than those of the wild-type strain, suggesting that MexCD-OprJ may be extruding HHQ, affecting the progressive intracellular accumulation of this signal. Since the expression of the *pqsABCDE-phnAB* operon, responsible of AQs biosynthesis (Gallagher et al., 2002), is activated in presence of PQS/HHQ (Wade et al., 2005; Hazan et al., 2010; Rampioni et al., 2016), we postulate that HHQ extrusion by MexCD-OprJ could be the main cause for the lower production of AQs observed in the *nfxB^∗^* strain, ultimately resulting in a defective QS-system.

### Overexpression of MexCD-OprJ Produces Minor Effects in the Synthesis of 3-oxo-C12-HSL and C4-HSL Autoinducers

**FIGURE 4:**
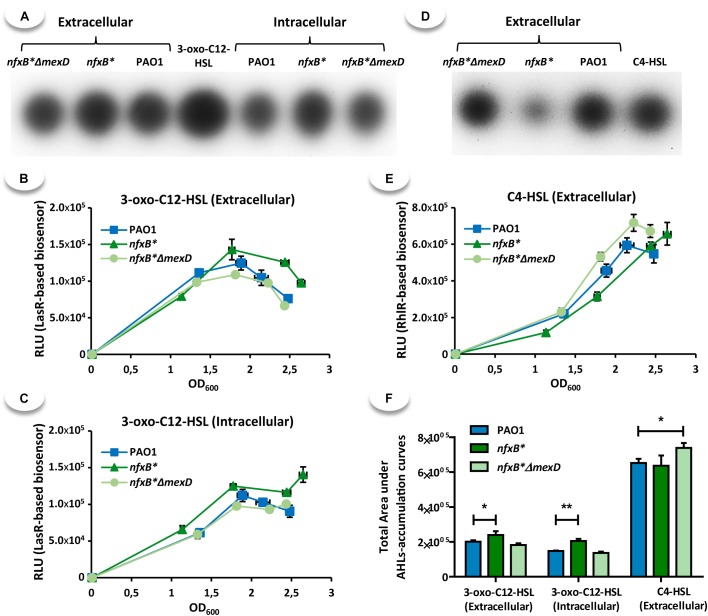
The *nfxB^∗^* mutant displays minor alterations in the kinetic of accumulation of 3-oxo-C12-HSL autoinducer. TLCs **(A,D)** and time course accumulation assays **(B,C,E)** were used to determine the accumulation of either 3-oxo-C12-HSL or C4-HSL autoinducer compounds. The samples for the TLC assays were extracted from cultures in late exponential phase (OD_600_ = 1.7) and the samples for the time course assay were taken at different time along the cell cycle (4, 5, 6, and 7 h post-inoculation). The area under the curve of each time course assay was calculated **(F)** and statistical significances were evaluated by using a Student’s two-tailed test with a confidence interval of 95% (^∗^*P* < 0.05; ^∗∗^*P* < 0.01; ^∗∗∗^*P* < 0.001). As shown, the overexpression of MexCD-OprJ has a slightly but significant effect on 3-oxo-C12-HSL accumulation. The *nfxB*^∗^ strain presented higher levels of 3-oxo-C12-HSL than PAO1 and *nfxB*^∗^Δ*mexD* both outside **(A,B,F)** and inside the cells **(A,C,F)**. In contrast, the supernatant accumulation of C4-HSL in late exponential phase was lower in the MexCD-OprJ overexpressing mutant as compared with PAO1 and *nfxB*^∗^Δ*mexD* strains **(D,E)**. However, the quantification of total area under the curves **(F)** only showed a significant increase in *nfxB*^∗^Δ*mexD* strain respect to both PAO1 and *nfxB^∗^*.

Since the Las, Rhl, and Pqs regulation systems are highly interconnected (McKnight et al., 2000; Diggle et al., 2003; Kasper et al., 2016), we wanted to know whether or not the excessive HHQ extrusion through MexCD-OprJ in the *nfxB*^∗^ mutant could be also affecting the production of the QS signals, 3-oxo-C12-HSL (autoinducer signal for Las system) and C4-HSL (autoinducer signal for Rhl system). As shown in Figures 4A–C,F, both intracellular and extracellular amounts of 3-oxo-C12-HSL are slightly higher in *nfxB^∗^* cultures than in either the wild-type PAO1 strain or the *nfxB*^∗^Δ*mexD* mutant. The opposite effect was observed for C4-HSL; the *nfxB^∗^* mutant accumulates slightly lower extracellular levels of this QS signal during late exponential phase (Figure 4D), reaching the levels of extracellular accumulation observed in both PAO1 and *nfxB*^∗^Δ*mexD* in early stationary phase (Figures 4E,F). This variation may also exist inside the cell due to the ability of C4-HSL to freely diffuse through cytoplasmic membrane (Pearson et al., 1999). Altogether, these results indicate that overexpression of MexCD-OprJ leads to minor alterations of AHLs production. These changes might be due to the strongly impaired production of PQS and HHQ.

### MexCD-OprJ Is Able to Extrude Kynurenine but Not Anthranilate, Both Precursors of AQs Signals

Our results indicate that the impaired QS response associated to the overexpression of the MexCD-OprJ efflux pump is mainly caused by a decreased production of PQS and HHQ, likely due to an excessive HHQ extrusion through this efflux system. The MexEF-OprN efflux pump is able to extrude both HHQ and its precursor kynurenine (Lamarche and Déziel, 2011; Olivares et al., 2012); extrusion of the latter is the main cause for the impaired QS response observed in MexEF-OprN overproducer strains (Olivares et al., 2012). A similar situation might also apply to MexCD-OprJ.

**FIGURE 5:**
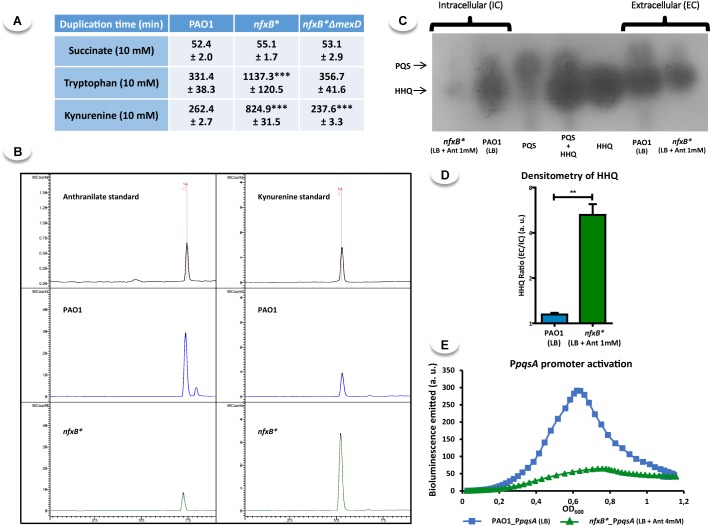
Impaired intracellular accumulation of anthranilate produced by an excessive kynurenine extrusion through MexCD-OprJ is not the cause for lower AQs production of the *nfxB*^∗^ mutant. Statistical significances were evaluated by using a Student’s two-tailed test and considered significant if *P* < 0.05, with a confidence interval of 95% (^∗^P < 0.05; ^∗∗^P < 0.01; ^∗∗∗^P < 0.001). **(A)** The duplication time of PAO1, *nfxB^∗^*, and *nfxB*^∗^Δ*mexD* strains growing in minimal medium with succinate (control), tryptophan or kynurenine (both anthranilate precursors) as sole carbon sources was determined. As shown, the *nfxB^∗^* mutant presents an impaired growth in both tryptophan and kynurenine, and deletion of *mexD* gene restored the growth rate in *nfxB^∗^* mutant, suggesting these compounds might be substrates of MexCD-OprJ. **(B)** Anthranilate and kynurenine accumulation in cell-free supernatants was quantified by HPLC-MS from PAO1 and *nfxB^∗^* cultures grown along 24 h in M63 minimal medium with succinate (10 mM) and tryptophan (10 mM) as sole carbon sources. Left panels anthranilate, right panels kynurenine. As shown, the supernatants from *nfxB^∗^* cultures contained more kynurenine and less anthranilate than those from the wild-type PAO1 strain, indicating that kynurenine is a substrate of MexCD-OprJ and anthranilate is not extruded by this efflux pump. **(C)** The production of AQs in PAO1 and *nfxB^∗^* strains growing in LB medium supplemented with anthranilate 1 mM was analyzed in early stationary phase (OD_600_ = 2.5) by TLC. **(D)** The extracellular vs. intracellular HHQ ratios were calculated measuring each one of the HHQ spots obtained in the TLC-assays by densitometry. **(E)** Real-time *pqsABCDE* expression was analyzed in both PAO1 and *nfxB^∗^* strains growing in LB medium and LB supplemented with anthranilate 4 mM respectively, using a chromosomal insertion of the reporter construction P*pqsA*::*luxCDABE.* The results show that anthranilate supplementation of LB medium does not restore the AQs production in the *nfxB*^∗^ strain **(C,E)**, reinforcing our hypothesis that HHQ extrusion **(D)** rather than kynurenine extrusion through MexCD-OprJ is the main cause for the QS-defective response of the *nfxB*^∗^ strain.

One of the immediate precursors of AQs in *P. aeruginosa* is anthranilate, which can < be synthetized either by PhnAB from chorismic acid or by kynurenine pathway when tryptophan is present in the medium (Deziel et al., 2004; Farrow and Pesci, 2007). Since the kynurenine pathway is the main source of anthranilate for AQs production when bacteria grow in rich LB medium (Farrow and Pesci, 2007), it could be possible that extrusion of some of the biosynthetic intermediates through MexCD-OprJ might affect the AQs production in *nfxB*^∗^. To test this hypothesis, we first analyzed the growth kinetic of the strains PAO1, *nfxB*^∗^, and *nfxB*^∗^Δ*mexD* in minimal medium containing tryptophan, kynurenine, or succinate as the sole carbon source. As shown in Figure 5A, the *nfxB*^∗^ mutant presents a growth defect in both tryptophan or kynurenine as the sole carbon source when compared to PAO1. These results strongly suggest extrusion of one or more intermediates of the kynurenine pathway through the MexCD-OprJ efflux system. As shown (Figure 5A) deletion of *mexD* in this mutant was enough to restore the wild-type growth rate, indicating that the observed growth defects, and the potential extrusion of these intermediates was solely due to the activity of MexCD-OprJ.

To analyze this possibility, we looked for the presence of kynurenine and anthranilate in the supernatants of PAO1 and *nfxB*^∗^ cultures. We observed a lower amount of anthranilate and a higher accumulation of kynurenine in the supernatants of *nfxB*^∗^ cultures (Figure 5B). Altogether, these results show that MexCD-OprJ is able to extrude kynurenine, but not anthranilate.

### The Low Levels of PQS and HHQ Observed in the *nfxB*^∗^ Strain Is Not Just Due to Kynurenine Extrusion

Having established that the constitutive overexpression of MexCD-OprJ efflux pump leads to a decrease in the extracellular accumulation of anthranilate, we wondered whether a low intracellular availability of anthranilate could be the cause of the impaired PQS and HHQ production observed in the *nfxB*^∗^ strain. To address this possibility, we grew PAO1 and *nfxB^∗^* in LB medium supplemented with 1 mM anthranilate and analyzed the production and accumulation of these two signals. As shown in Figure 5C, anthranilate supplementation does not restore PQS/HHQ production to wild-type levels in the *nfxB^∗^* strain. In addition, our results indicate that the *nfxB^∗^* strain continues to extrude HHQ at higher levels than those observed in the wild-type strain under these conditions (Figure 5D).

We entertained the possibility that a higher anthranilate concentration was needed to restore AQs production to wild-type levels in *nfxB*^∗^. To this end, we supplemented LB medium with up to 4 mM anthranilate and analyzed the activation of the *pqsABCDE* promoter in real-time in both PAO1 and *nfxB*^∗^. As shown in Figure 5E, a higher concentration of anthranilate did not restore the activation of the *pqsABCDE* promoter in the *nfxB^∗^* strain. These results indicate that a low anthranilate concentration caused by kynurenine extrusion is not the main underlying cause for the impaired PQS and HHQ production observed in this strain. These results further support the notion that an excessive, non-physiological, extrusion of HHQ caused by the overexpression of MexCD-OprJ is likely the main cause for the lower accumulation and production of HHQ and PQS in the multidrug resistant *nfxB^∗^* mutants.

### The Low Production of AQs Associated to MexCD-OprJ Overexpression Is Not Caused by an Impaired Intracellular Accumulation of Octanoate

Octanoate is the other direct precursor of PQS and HHQ (Dulcey et al., 2013). Once we established that anthranilate synthesis is not the limiting step in the production of AQs by the *nfxB^∗^* strain, we wondered whether a hypothetical low production or intracellular accumulation of octanoate might be affecting the AQs production in this strain. For that purpose, we measured the progressive accumulation of AQs in both cell-free supernatants and cellular extracts from PAO1, *nfxB^∗^*, and *nfxB*^∗^Δ*mexD* cultures grown in LB supplemented with 5 mM octanoate.

**FIGURE 6:**
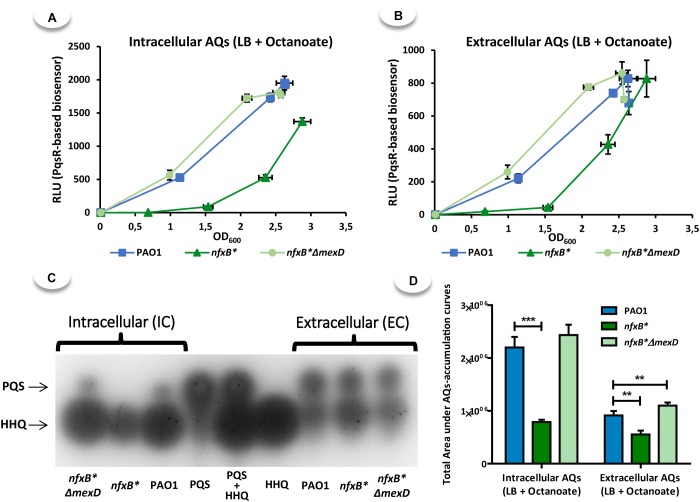
LB supplementation with octanoate increased the AQs production in PAO1, *nfxB^∗^*, and *nfxB*^∗^Δ*mexD* strains but, in the case of *nfxB^∗^*, but the accumulation levels remain being lower in *nfxB^∗^* than observed in the other strains. To determine the time course production of AQs in PAO1, *nfxB^∗^*, and *nfxB*^∗^Δ*mexD* strains, we extracted these compounds from both the cells **(A)** and the cell-free supernatants **(B)** at different times along the cell cycle (4, 5, 6, and 7 h post-inoculation). Additionally, the last points of time course extractions were analyzed by TLC **(C)** in order to know the proportion of PQS and HHQ present on each AQs-extracts. The total area under each time course accumulation curve was quantified **(D)** and statistical significances were evaluated by using a Student’s two-tailed test and considered significant if *P* < 0.05, with a confidence interval of 95% (^∗^*P* < 0.05; ^∗∗^*P* < 0.01; ^∗∗∗^*P* < 0.001). The results show that supplementation of LB with 5 mM octanoate, even allowing *nfxB*^∗^ strain to accumulate levels of AQs out of the cells near to those in PAO1 and *nfxB*^∗^Δ*mexD* **(B–D)**, was insufficient to restore the intracellular accumulation of PQS and HHQ **(A,C,D)**. Furthermore, the fact that in TLC assay **(C)**, the spot corresponding with HHQ present in *nfxB*^∗^ supernatant is slightly higher than that in PAO1 and *nfxB*^∗^Δ*mexD*, together with the evident low intracellular accumulation of HHQ in the *nfxB*^∗^ strain, confirm our hypothesis that MexCD-OprJ is able to extrude HHQ.

**FIGURE 7:**
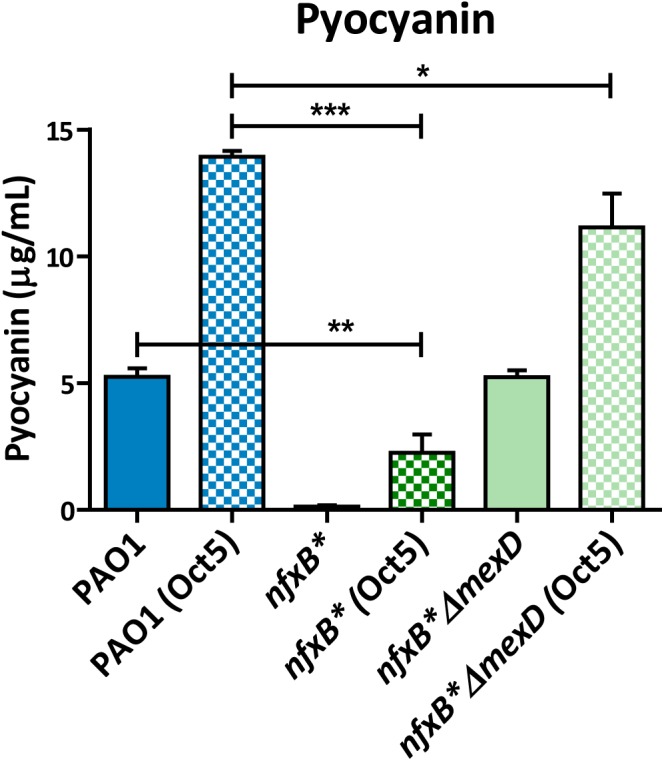
LB supplementation with octanoate increased the pyocyanin production in all analyzed strains, but *nfxB^∗^* remained producing lower levels than those observed in PAO1 when grew in LB medium. For pyocyanin assay, the strains were grown in LB medium with or without octanoate (5 mM) along a time lapse of 20 h and the pyocyanin was extracted with chloroform-based protocol as is described in Methods. Statistical significances were evaluated by using a Student’s two-tailed test and considered significant if *P* < 0.05, with a confidence interval of 95% (^∗^*P* < 0.05; ^∗∗^*P* < 0.01; ^∗∗∗^*P* < 0.001). The results show that supplementation of LB with 5 mM octanoate, even allowing *nfxB*^∗^ strain increase the pyocyanin production, was insufficient to reach the levels produced by PAO1 in LB without octanoate. This indicate that a hypothetical low intracellular accumulation of octanoate is not the main cause for defective pyocyanin production observed in *nfxB*^∗^ strain.

In agreement with previous findings (Dulcey et al., 2013), we found that the intracellular accumulation of AQs (Figure 6A) and pyocyanin production (Figure 7) increase when octanoate is added. However, these increases were similar in all strains, and both the pyocyanin production and the absolute AQs levels reached inside cells were still lower in *nfxB*^∗^ than in PAO1 or in *nfxB*^∗^Δ*mexD*. These results indicate that a lower availability of octanoate is not the cause of the impaired QS response displayed by the *nfxB*^∗^ strain.

It is worth mentioning that, although the *nfxB^∗^* supernatants exhibit a delay in AQs accumulation in the presence of 5 mM octanoate, the supernatants from all three strains exhibit similar levels when the cultures reach high cell densities (OD_600_ > 2.5) (Figure 6B). In contrast, the intracellular AQs accumulation remains lower in the *nfxB^∗^* strain (Figure 6A). In the same way, the quantification of the areas under the curve for AQs-accumulation shows that the differences observed in these strains are lower in the supernatants when compared to cell extracts (Figure 6D). Further, the analysis by TLC of AQs extracted from the last point of the time course assay showed that, while the intracellular accumulation of PQS and HHQ remained being lower in the case of *nfxB*^∗^, the extracellular accumulation of these two AQs were similar among PAO1, *nfxB*^∗^ and *nfxB*^∗^Δ*mexD* cultures (Figure 6C). These results further reinforce the hypothesis that MexCD-OprJ is able to extrude HHQ (and likely PQS as well), and confirm that overexpression of this system adversely affects the intracellular accumulation of AQs. This extrusion can be considered the bottleneck that precludes a proficient PQS production as well as the onset of a proper QS response in *nfxB^∗^*-type mutants.

## Discussion

In the current work, we demonstrate that a *P. aeruginosa nfxB^∗^* mutant, which overexpresses the MexCD-OprJ efflux pump, exhibits an impaired QS response due the extrusion of HHQ. Specifically, this non-physiological extrusion leads to a decrease in the expression of the *pqsABCDE* operon responsible for AQs synthesis, which affects AQs-dependent and the PqsE-dependent regulons that comprise the genes involved in swarming motility, and in the production of pyocyanin, rhamnolipids, and proteases among others (Hazan et al., 2010; Rampioni et al., 2010, 2016).

The QS response in *P. aeruginosa* consists mainly on the Las, Rhl, and Pqs systems which are dependent on the 3-oxo-C12-HSL, C4-HSL, and PQS/HHQ autoinducers, respectively (Williams and Camara, 2009). The cross-regulation between these QS-systems is hierarchically understood, with the Las system located at the top, activating the other two QS systems, and followed by the Pqs-dependent activation of the Rhl-system, and by the Rhl-dependent repression of the Pqs-system (Dubern and Diggle, 2008; Williams and Camara, 2009). However, evidence exists that the hierarchy and the relationship between these QS-systems may be modulated depending on environmental conditions and the activity of global regulators as MvaT or RsmA among others (Choi et al., 2011; Mellbye and Schuster, 2014; Hammond et al., 2016; Maura et al., 2016; Welsh and Blackwell, 2016). In addition, recent studies have highlighted the relevant role of the feedback-regulation between Las, Rhl, and Pqs systems as well as the relevance of the PqsE and RhlR regulators (Deziel et al., 2005; Farrow et al., 2008; Dekimpe and Deziel, 2009; Hazan et al., 2010; Rampioni et al., 2010, 2016). Indeed, it has been demonstrated that the expression of approximately 90% of the genes in the AQs-regulon may be regulated through *pqsE* induction (Hazan et al., 2010). Likewise, the non-virulent phenotype prompted by the absence of AQs synthesis may be by-passed through *pqsE* induction, restoring the full *P. aeruginosa* virulence (Deziel et al., 2005; Hazan et al., 2010; Rampioni et al., 2010). Further, the regulation of several QS-dependent factors could be redundant. In such a way, the production of elastase, rhamnolipids, or pyocyanin, which are mainly under the control of Las, Rhl, and Pqs systems, respectively, are also regulated by PqsE independently of AQs production (Deziel et al., 2005; Hazan et al., 2010; Rampioni et al., 2010, 2016). In addition, expression of *rhlR* increases upon *pqsE* induction at the same time that some functions of PqsE as a QS-regulator are dependent on RhlR and C4-HSL production, thus establishing a complex feedback regulation loop (Hazan et al., 2010). Even more, exogenous addition of C4-HSL to the cultures may partially complement some of the phenotypes impaired in a *pqsE* mutant, such as pyocyanin production (Farrow et al., 2008; Hazan et al., 2010; Rampioni et al., 2010). Recent work indicates that PqsE is an alternative ligand synthase and PqsE and RhlR function as a QS-autoinducer synthase–receptor pair able of regulate the *P. aeruginosa* QS response independently of RhlI (Mukherjee et al., 2018).

Given this role of PqsE as one of the main elements in the QS regulatory network, we postulate that a reduced production of PQS and HHQ, together with a decreased *pqsE* expression, are the main causes for the lack of QS-response associated to the constitutive overexpression of the MexCD-OprJ efflux system. In this work, we show that expression of QS-regulated genes decreases in an *nfxB*^∗^ antibiotic resistant mutant and that inactivation of the MexCD-OprJ efflux pump in this background restores expression of these genes to wild-type levels, being even higher in some cases (Figure 2). Similar results were obtained with some QS-regulated phenotypes such as the production of elastase, protease IV, pyocyanin, rhamnolipids, and swarming motility (Figure 1), indicating that the alterations in the QS-response displayed by the *nfxB^∗^* mutant are directly caused by the increased expression and activity of the MexCD-OprJ efflux system. We also demonstrated that loss of function of NfxB leads to an excessive extrusion of HHQ through the overexpressed MexCD-OprJ efflux pump, resulting in a low intracellular accumulation. Expression of *pqsABCDE* during exponential and early stationary growth phases is subjected to a positive feed-back transcriptional regulation under the control of the PqsR-(PQS/HHQ) complex (Rampioni et al., 2016). Therefore, the non-physiological HHQ extrusion through MexCD-OprJ may abrogate this positive feed-back regulation and directly cause the decrease in *pqsABCDE-phnAB* expression (Figure 2) and the AQs synthesis impairment (Figure 3) observed in the *nfxB*^∗^ mutant. We also showed that this defective AQs accumulation could not be restored by adding either anthranilate or octanoate, the two PQS/HHQ main precursors (Figures 5, 6), reinforcing the concept that the main cause for the defective QS-response associated to *nfxB* mutations is an excessive extrusion of HHQ through MexCD-OprJ, and not of metabolic precursors as kynurenine, also extruded by MexCD-OprJ. Additionally, the presence of similar levels of PQS in the supernatants of PAO1, *nfxB*^∗^ and *nfxB*^∗^Δ*mexD* growing in presence of octanoate, together with the absence of this autoinducer signal in the cell-extracts of *nfxB*^∗^ (Figure 6C) suggests that PQS could also be a MexCD-OprJ substrate.

To sum up, here we show that the AQs production is affected by the increased efflux of HHQ by the MexCD-OprJ RND system overexpressed in the *nfxB*^∗^ ciprofloxacin-resistant mutants. To note here that this type of antibiotic resistant mutants that are isolated *in vivo* from ciprofloxacin-treated patients (Jeannot et al., 2008). As a consequence, expression of the Pqs-regulon, which also comprises those PqsE-regulated genes in a PQS-independent way (Rampioni et al., 2010, 2016), is strongly altered in an *nfxB*^∗^ mutant. This alteration may have minor collateral effects on the AHLs-dependent QS systems and is likely the main cause for the low virulence profile observed in antibiotic resistant mutants overproducing MexCD-OprJ.

Previous work has shown that the *P. aeruginosa* MDR efflux pump MexEF-OprN is able of extruding kynurenine and HHQ as well (Lamarche and Déziel, 2011; Olivares et al., 2012). However, different to the situation with MexCD-OprJ, the reason for the impairment in the QS response of a MexEF-OprN overexpressing mutant was mainly the extrusion of kynurenine (Olivares et al., 2012), the HHQ precursor. Similarly, both efflux pumps can accommodate the same antibiotics, although the affinity for each of them can be different. Indeed, overexpression of whatever these two efflux systems increases ciprofloxacin and chloramphenicol resistance but at different levels, being MexCD-OprJ more efficient extruding ciprofloxacin and MexEF-OprN extruding chloramphenicol (Linares et al., 2005). It has been shown that, in addition to contributing to a coordinated response of the bacterial population, several QS signal molecules (Williams, 2007; Pacheco and Sperandio, 2009; Martinez, 2014) are also involved in inter-specific communication. For example, it has been shown that AQs may function as antimicrobial compounds against *Staphylococcus aureus*, a bacterial species commonly detected together *P. aeruginosa* in polymicrobial infections (Mashburn et al., 2005; Nguyen et al., 2015; Nguyen and Oglesby-Sherrouse, 2016). Further, HHQ also is able to induce apoptosis in human mesenchymal stem cells (Holban et al., 2014), and to impair the production of several factors implicated in the innate immune response affecting the binding of the nuclear factor-κβ to its targets (Kim et al., 2010). The fact that in this work we demonstrate that MexCD-OprJ is able to extrude HHQ, altering the accumulation level of the autoinducer signals produced by *P. aeruginosa*, opens a new perspective over the potential functions of this RND efflux system in the interactions between this opportunistic pathogen and other co-existing species (including the human host); a hypothesis that remain to be explored.

The activation of the QS-response implies an increase in expression of 100s of genes, and it has been estimated that this consumes approximately 10% of *P. aeruginosa* metabolic resources (Haas, 2006). Under this panorama, QS-defective mutants, which are unable to produce different exoproducts such as siderophores or proteases relevant for nutrients uptake, could be cheaters supported by neighbor bacteria able to produce these QS-dependent factors (Wilder et al., 2011; Pollak et al., 2016). The evolution of *P. aeruginosa* along the chronic infection of the lungs of cystic fibrosis patients involves a radiative evolution, with different morphotypes, including QS-deficient mutants, co-existing in the lung of each patient (LaFayette et al., 2015). It is conceivable that the mutants lacking a proficient QS response remain in the population because they can obtain the benefits brought about by an appropriate QS response carried out by co-existing bacteria without the cost associated with it. In agreement with this hypothesis, it might be possible that antibiotic resistance mediated by MexCD-OprJ overproduction allows *nfxB^∗^* mutants to function as cheaters in the mixed populations colonizing the lung of the infected patient. This feature may have important implications concerning the persistence of antibiotic resistant mutants even in the absence of selection (Knoppel et al., 2017).

## Author Contributions

MA-R performed the experimental work. JO-P and JM designed the study. MA-R, JO-P, MC, CA-O, and JM contributed to the interpretation of the results and in writing the article.

## Conflict of Interest Statement

The authors declare that the research was conducted in the absence of any commercial or financial relationships that could be construed as a potential conflict of interest.
